# Entropic Control of an Excited Folded-Like Conformation in a Disordered Protein Ensemble

**DOI:** 10.1016/j.jmb.2018.06.008

**Published:** 2018-06-07

**Authors:** Sneha Munshi, Divya Rajendran, Athi N. Naganathan

**Affiliations:** Department of Biotechnology, Bhupat & Jyoti Mehta School of Biosciences, Indian Institute of Technology Madras, Chennai 600036, India

**Keywords:** rough landscape, thermodynamics, dynamics, proline, multi-probe spectroscopy

## Abstract

Many intrinsically disordered proteins switch between unfolded and folded-like forms in the presence of their binding partner. The possibility of a pre-equilibrium between the two macrostates is challenging to discern given the complex conformational landscape. Here, we show that CytR, a disordered DNA-binding domain, samples a folded-like excited state in its native ensemble through equilibrium multi-probe spectroscopy, kinetics and an Ising-like statistical mechanical model. The population of the excited state increases upon stabilization of the native ensemble with an osmolyte, while decreasing with increasing temperatures. A conserved proline residue, the mutation of which weakens the binding affinity to the target promoter, is found to uniquely control the population of the minor excited state. Semi-quantitative statistical mechanical modeling reveals that the conformational diffusion coefficient of disordered CytR is an order of magnitude slower than the estimates from folded domains. The osmolyte and proline mutation smoothen and roughen up the landscape, respectively, apart from modulation of populations. Our work uncovers general strategies to probe for excited structured states in disordered ensembles, and to measure and modulate the roughness of the disordered landscapes, inter-conversion rates of species and their populations.

Intrinsically disordered proteins (IDPs) sample multiple conformations in equilibrium whose populations and inter-conversion rates are highly sensitive to ambient conditions enabling tight control [1–3]. Given the complex and dynamic underlying landscape, the role of such conformations in binding equilibria and the energetic and entropic origins that determine the nature of sub-ensembles has been difficult to disentangle [4–6]. However, given that the unfolded states of many folded proteins exhibit predominantly native-like contacts [7, 8], it is possible that molten-globular proteins and IDPs also sample partially structured or binding competent states in the absence of the binding partner, a feature that can influence binding affinity and promiscuity through different mechanisms [4, 9–13]. Proline residues can have complex and context-dependent effect on stability, kinetics and function in both ordered and disordered proteins [14–20]. Specifically, the enrichment and conservation of proline residues in IDPs raises questions on whether the minimal backbone conformational entropy of proline can determine populations and hence function.

In this study, we examine the conformational behavior of the DNA binding domain of CytR (termed CytR from hereon) that is uniquely disordered compared to other well-folded members of the LacR family [21]. CytR exists in dynamic equilibrium with numerous sub-states while exhibiting large enthalpic fluctuations despite undergoing a collapse transition at higher temperatures (>303 K) [22]. Through a combination of multi-probe equilibrium spectroscopy, kinetics by monitoring a natural tyrosine (Y53) and theoretical modeling, we show that disordered CytR samples an excited folded-like conformation in a temperature-dependent manner. Remarkably, we find that a single conserved proline residue, P33, determines the population of this minor state; the mutation P33A results in non-trivial changes in equilibrium, kinetics and unexpectedly in even the binding with cognate DNA.

CytR adopts a three-helix bundle structure in the presence of DNA (Fig. 1A) while being disordered otherwise (blue in Fig. 1B). The far-UV CD thermal unfolding curve in the absence of DNA points to a loss of secondary-structure with temperature and that is abrogated in the presence of urea (blue in Fig. 1B, C). In other words, if CytR were completely unfolded, it would exhibit a spectrum or a melting profile similar to that observed in the presence of 6 M urea (black in Fig. 1B and C). The melting of secondary structure by far-UV CD (blue in Fig. 1C) therefore suggests that the disordered ensemble of CytR samples helical-like conformations at low temperatures and that are lost at higher temperatures. The C-terminal region of the third helix harbors a tyrosine (Y53) that makes specific long-range interactions with the region following first helix in the folded-form of CytR (i.e., in the presence of DNA; Fig. 1A). Near-UV CD spectral analysis should therefore serve as a strategic probe for monitoring the folded form or a conformation that is folded-like, that is, making a long-range contact between first and third helices, in the disordered ensemble. Near-UV CD experiments indeed point to a loss of excitonic coupling associated with buried tyrosine as a function of temperature, suggesting that the tertiary environment is distinctly modulated (blue in Fig. 1D) apart from the secondary structure. Since near-UV CD is a powerful probe for tertiary structure, the presence of tyrosine signal is compelling evidence that the unfolded ensemble populates specific, long-lived and well-structured conformations. Control experiments at 6 M urea indicate little changes in the near-UV CD signal (black in Fig. 1D), highlighting that the changes observed in the absence of urea (blue in Fig. 1C, D) potentially arise from folded-like sub-populations.

The quantum yield (QY) of Y53 decays near-linearly with temperature in the presence of urea (black in Fig. 2A) but displays a weak sigmoidal unfolding curve under native conditions (blue in Figs. 2A and S1 in the Supporting Information). Unlike near-UV CD, QY measurements are confounded by the intrinsic temperature dependence of fluorescence. However, kinetic amplitudes are free of such effects, and hence, we resorted to stopped-flow kinetic experiments to extract the time-scales and amplitudes of structural changes. Stopped-flow kinetic experiments were performed by diluting out the protein in denaturant solution (say, 4 or 6 M urea) to native-like conditions (~ 0.36 or ~ 0.54 M urea and pH 7.0, 20 mM phosphate buffer) at different temperatures. Single-exponential relaxation phases are observed at all temperatures with the relaxation rates (*k*_obs_) surprisingly ranging from just ~ 30 s^−1^ at 283 K to ~ 150 s^−1^ at 303 K (Fig. 2B, D). The amplitude of the single-exponential phase decreases with temperature signifying a loss in the population of a signal-competent state (Fig. 2C). The kinetic amplitudes are in very good agreement with estimates from equilibrium, indicating that there are no missing amplitudes (Fig. 2C). Interestingly, the *k*_obs_ displays a steeper dependence with temperature compared to the expectation from changes in solvent viscosity alone (Fig. 2D).

There are two potential scenarios that contribute to decreasing amplitude with temperature—signal changes could arise from conformational redistributions in the folding direction or in a direction that is orthogonal to it. In the former, a small fraction of folded-like conformation exists in the unfolded ensemble (a minor excited state) whose population is lost with increasing temperatures. In the latter, the relaxation represents an inter-conversion between two unfolded-like conformational sub-states with different tyrosine environments contributing to the observed signal change in equilibrium and kinetics. While the latter scenario is unlikely, it is possible to distinguish them through experiments that modulate the stability of CytR. In other words, increasing or decreasing the stability of CytR should in turn increase or decrease the amplitude of the kinetic phase (which is a measure of the excited minor state population), respectively.

We sought to increase the stability of CytR ensemble by adding TMAO, a natural osmolyte. In addition to increasing the overall secondary and tertiary structure of CytR ensemble, TMAO also modulates the fluorescence of Y53 (green circles in Figs. 1 and 2) compared to experiments in the absence of TMAO (blue circles in Figs. 1 and 2). Note that the far-UV CD signal at 298 K approaches that of the folded homologs LacR and PurR (−15,000 deg. cm^2^ dmol^−1^; see Fig. 3A and Refs. [22, 23]). The implication is that the population of specific conformations within the CytR ensemble that exhibit distinct secondary and tertiary structure (i.e., the minor state) is proportionately increased on increasing the stability of the CytR ensemble (green circles in Figs. 1 and 2). Kinetic experiments reveal a single-exponential relaxation phase with a rate that is near identical to native conditions (Fig. 2D). The amplitudes increase by a factor of 2 at the lowest temperatures in the presence of TMAO while displaying a sigmoidal-like trend with temperature (inset to Fig. 2C). The increasing amplitude on stability increase is kinetic evidence that the signal-competent state, or equivalently the conformations whose population increases upon TMAO addition, is in the folding direction. Moreover, this minor state has to have a conformation similar to the fully folded state since the tyrosine signal arises from specific interactions between the first and third helices (and which is absent in 6 M urea; Figs. 1D and 2A). These observations are consistent with theoretical predictions from a statistical model [23], all-atom MC simulations [22] and more specifically with heteronuclear single quantum coherence–NMR experiments [21]. The latter reveals additional backbone resonances (87 resonances compared to the expected 63 non-proline amino acids) in the unbound form of CytR hinting at an alternate conformation in the slow-exchange regime.

The corollary to above experiments is that any destabilization of the disordered ensemble should contribute to lower population of the minor excited state and hence lower kinetic amplitudes compared to the WT. A plausible candidate is the trans-proline at position 33 (P33, present in the loop that connects the second and third helices; Fig. 1A) that is not present in other LacR family members (Supporting Fig. S2). We hypothesize that the structural changes in the unfolded ensemble are a manifestation of the modulation of long-range order brought about by P33 that entropically brings the helices together through its backbone rigidity. We indeed find that P33A mutant displays lower “stability,” secondary and tertiary structure (very similar to that of urea but in native buffer conditions) and smaller kinetic amplitude that decays rapidly with temperature compared to the WT (red circles in Figs. 1 and 2). Thus, a small destabilization of the unfolded ensemble dramatically affects the conformational properties with the effect felt nearly 16 Å away (C_α_ distance between P33 and Y53) as observed by near-UV CD and fluorescence of Y53. The rate of inter-conversion between the unfolded and folded-like conformations is marginally slower (red in Fig. 2D), implying that either the underlying landscape has roughened up (a dynamic effect or decrease in landscape roughness from a decrease in *D*_eff_) or the free energy barrier has increased (a thermodynamic effect, an increase in Δ*G*^†^) as expected from the equation *k*_obs_ *= D*_eff_ exp(−Δ*G*^†^/*RT*) [24, 25].

It is challenging to distinguish between the two scenarios discussed above without constructing energy landscapes of disordered ensembles that also reproduce the equilibrium temperature dependence. We therefore resort to the Wako–Saitô–Muñoz–Eaton (WSME) model [26–28], employing an approach as before [23] but with sequence-based entropy (see Supporting Methods and Table T1). The WSME model is able to semi-quantitatively reproduce the unfolding curve of the WT as observed by far-UV CD employing the LacR unfolding curve as a reference [29] for the folded baseline (blue curve in Fig. 3A and Ref. 23). The effect of P33A mutation is *predicte*d by employing the same mean-field conformational entropy cost for A33 as other residues (red curve in Fig. 3A), instead of zero conformational entropic penalty employed for P33 in the WT (see Supporting Methods). The effect of TMAO is modeled by increasing the magnitude of the van der Waals interaction term that simultaneously matches the far-UV CD unfolding curve (green in Fig. 3A). The resulting one-dimensional (1D) free energy profiles as a function of the number of structured residues are flat and rough with multiple conformational sub-states as expected for disordered systems (Fig. 3B) and account for thermodynamic contributions to the rates. Marginal thermodynamic barriers separate the macroscopic states and they arise primarily as a result of projection onto a single order parameter [22].

The probability densities derived from the 1D profiles highlight an increase in the folded-state population with increasing stabilization of the disordered ensemble (Fig. 3B). The population of the folded-like conformation is predicted to be between ~ 8% under native conditions and at 283 K (Supporting Fig. S3). Diffusive calculations on the free energy profiles employing the discrete formulation of the 1D diffusion equation [30] and mimicking the experimental protocol reveal single-exponential kinetic phases (see Methods and Supporting Fig. S4) whose amplitude decreases with temperature (Fig. 3C). The Eigen vectors corresponding to the first non-zero Eigen value signal that the dominant contribution to the observed amplitude arises from an equilibration between the unfolded and folded-like macrostates (Fig. 3D). The relative ordering of amplitude among the three kinetic experiments (native buffer conditions for WT and P33A, and WT in TMAO) is also reproduced very well by our approach (Fig. 3C). It is important to note that the free energy profiles, fractional population and kinetic amplitudes are predictions from the model that takes in purely the equilibrium data as input.

To reproduce the precise magnitude of rates as a function of temperature, we employ a diffusion coefficient that is temperature dependent as observed in folded proteins, that is, *D*_eff_ *= k*_0_exp(−*E*_a_/*RT*), where *E*_a_ is the activation energy that includes internal friction effects and changes in solvent viscosity [25]. The populations derived from the model, in turn, account for the thermodynamics thus providing a well-constrained estimate of the dynamic term in the rate equation. On reproducing the rates by modulating *k*_0_ and *E*_a_ (Fig. 3E), we find that effective diffusion coefficient of disordered CytR is nearly an order of magnitude slower (blue in Fig. 3F) than that expected for folded proteins (circles in Fig. 3F) [31–33]. The *D*_eff_ speeds up on smoothening the landscape (with TMAO, green in Fig. 3F) and slows down upon introducing the P33A mutation (red in Fig. 3F). The *E*_a_ is estimated to be 46.3 kJ mol^−1^, which is effectively ~ 1 kJ mol^−1^ per residue when considering the structured domain of CytR alone (47 residues) consistent with kinetic studies on folded proteins [25].

Given the large differences in structural signatures, kinetics and populations on a single mutation, it is imperative to question the functional role of P33 in CytR. In this regard, we find that the binding affinity of P33A mutant to the natural *udp* promoter is significantly weakened at both low and higher temperatures compared to the WT (Fig. 4 and Supporting Methods), precluding a reasonable estimate of binding affinities. These experiments therefore show that P33 acts as a pivot to populate a folded-like pose enabling stronger DNA binding.

In summary, we show that the native ensemble of CytR, while being predominantly disordered, still populates an excited folded-like state whose population decreases with temperature. This is, to the best of our knowledge, the first such experimental observation in the field of IDPs. Modulation of solution conditions and mutation contribute to changes in kinetic amplitudes expected of an excited state that populates in the folding direction. Our experimental observations are also semi-quantitatively consistent with previous NMR experiments, atomic-level simulations and particularly with a statistical mechanical model allowing us to decouple dynamic and thermodynamic effects. We also show that the conformational diffusion coefficient of an IDP is just about an order of magnitude slower than that of folded proteins. A conserved proline at position 33 entirely controls the population of the excited state by acting as a pivot and entropically enabling the formation of a folded-like structure within the unfolded ensemble. Since binding to DNA is significantly weakened on mutating the proline, the minor state seems to be a pre-requisite for efficient binding to DNA. Given that a large number of IDPs fold upon binding their targets, probes that are sensitive to long-range structure could be engineered to explore for the presence of native-like or partially structured conformations in disordered ensembles.

## Supplementary Material

Supplementary data to this article can be found online at https://doi.org/10.1016/j.jmb.2018.06.008.

Supporting Information

## Figures and Tables

**Fig. 1:**
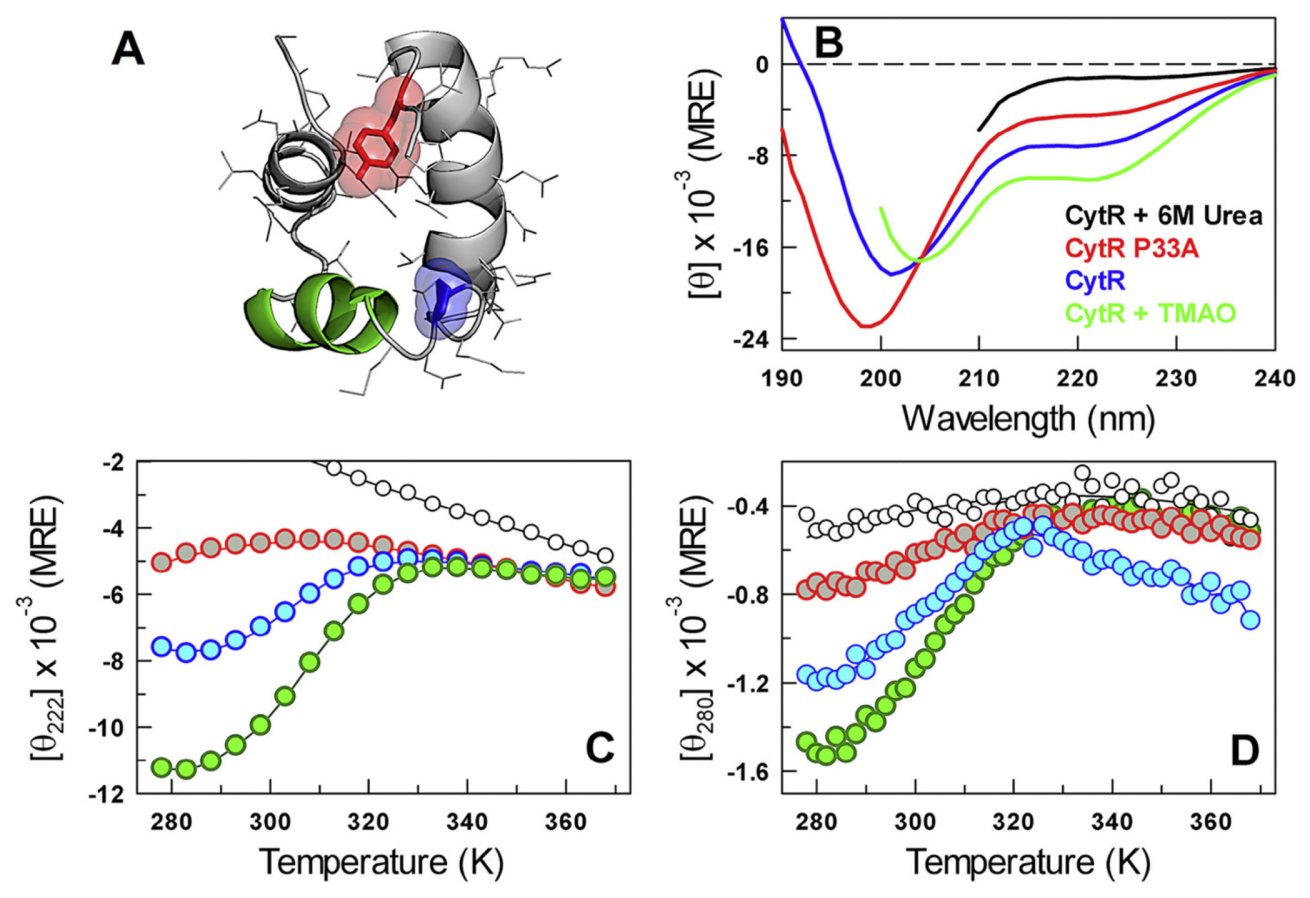
Equilibrium structural changes. (A) Structure of the DNA-bound conformation of CytR highlighting the DNA-binding second helix (green), P33 (blue) and Y53 (red). (B–D) The far-UV CD spectra (panel B; in MRE units of deg. cm^2^ dmol^−1^), far-UV CD unfolding curve (C) and the near-UV CD unfolding curve (D) of CytR and its variants following the color-code in panel B. Note that these experiments have been carried out in the absence of DNA.

**Fig. 2:**
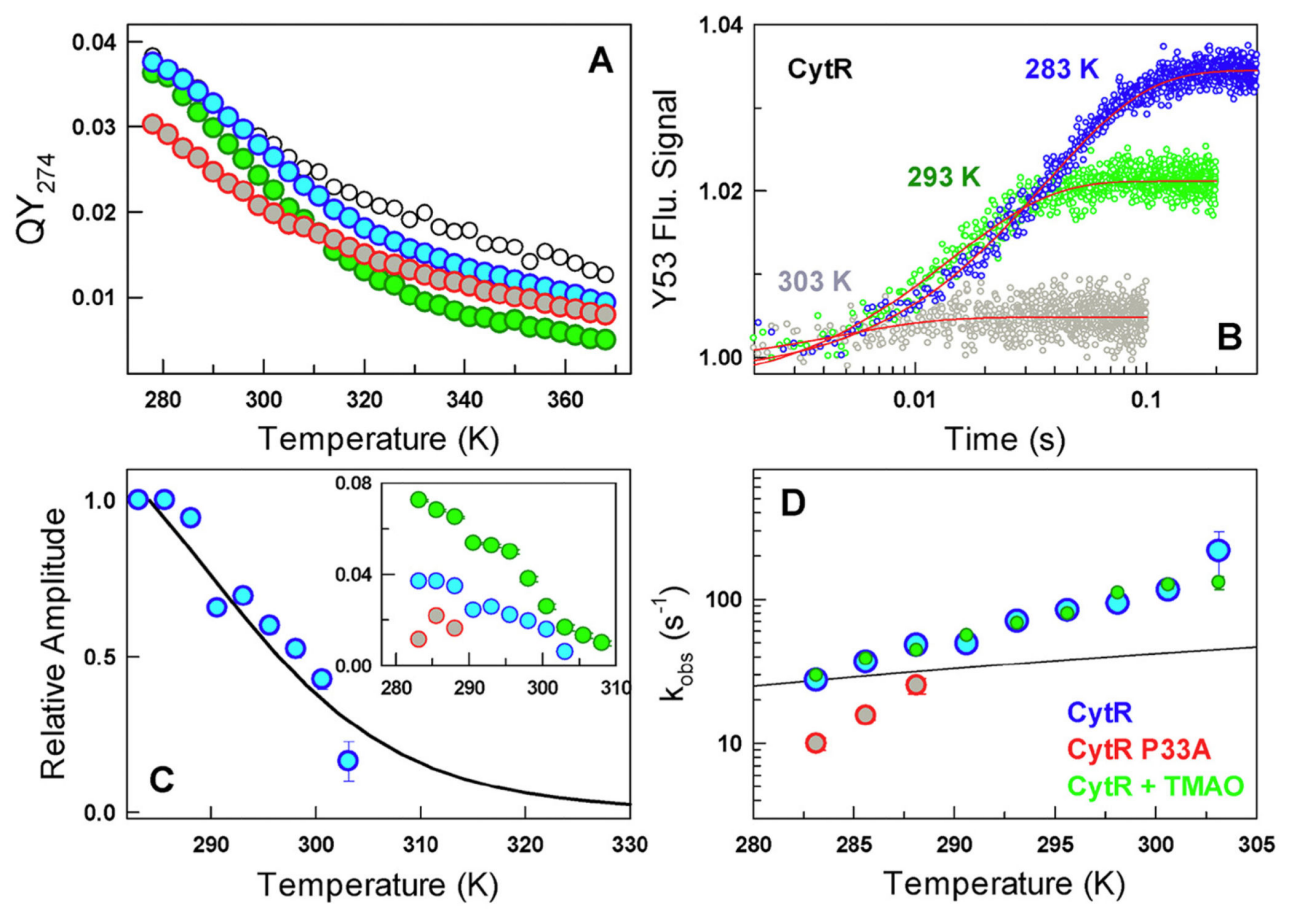
Kinetics and temperature dependence. (A) Temperature dependent QY of CytR (blue), CytR in 6 M urea (black), CytR + 0.5M TMAO (green) and CytR P33A (red). (B) Refolding kinetic traces of CytR from stopped-flow experiments (circles) and the single-exponential fits (red). (C) The normalized kinetic amplitude of CytR (circles) follows the changes in population expected from a two-state fit to the QY unfolding curve (black). Inset: Kinetic amplitudes as a function of temperature following the color code in panel A. Note that the amplitudes directly signal the relative populations of the minor state. (D) Observed rate constants under near-native conditions as a function of temperature (circles) together with the expectation from changes in solvent viscosity alone (black).

**Fig. 3:**
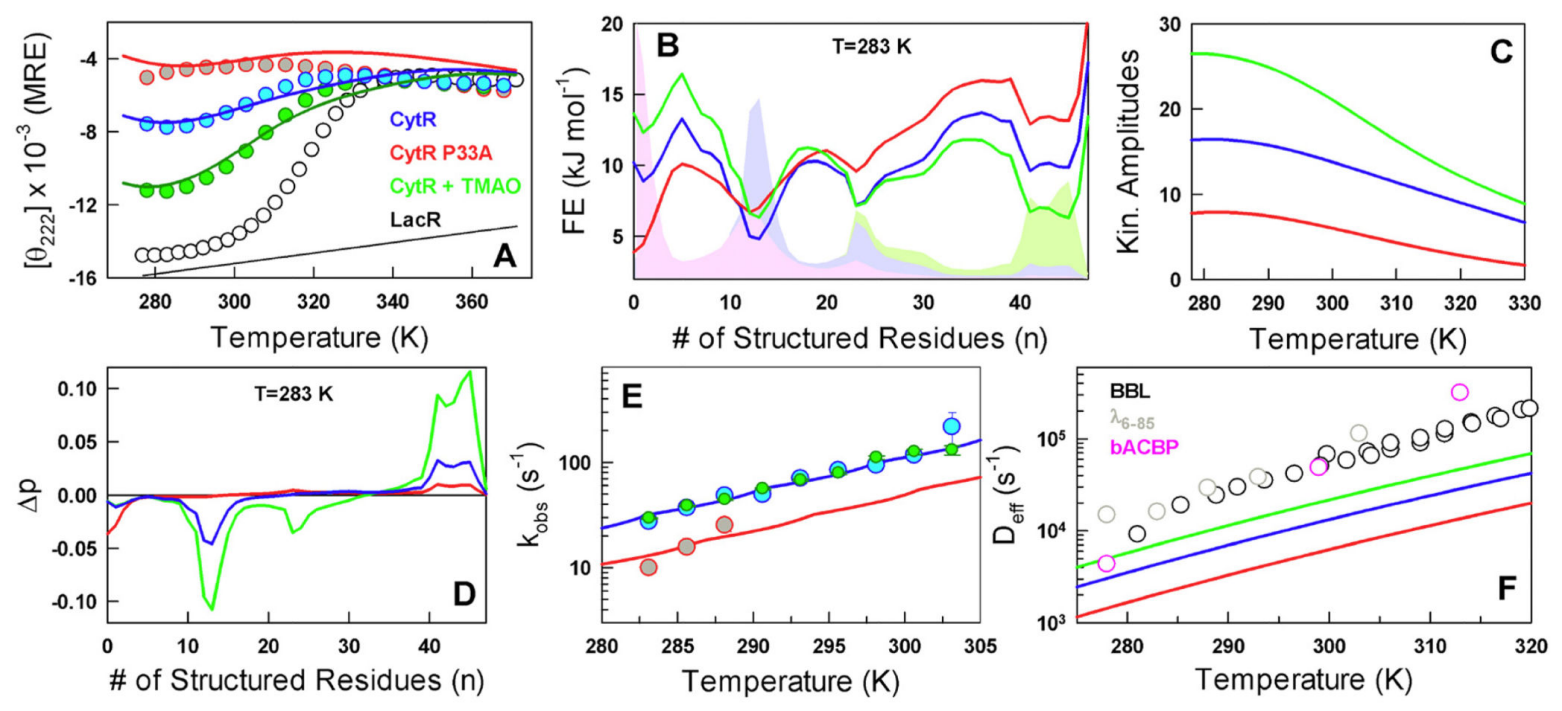
Modeling experimental signals and predictions. (A) Modeling equilibrium far-UV CD signal changes through the WSME model. The P33A unfolding curve is a prediction from the model. The LacR unfolding and its folded baseline are used as a reference. The color coding is maintained in all panels. (B) Predicted 1D free energy profiles following the color code in panel A. The corresponding probability distributions are shown in shaded areas. Note that the folded probability distribution increases with increasing stabilization of the disordered ensemble. (C) Predicted kinetic amplitudes as a function of temperature that mirror the relative ordering observed in experiments (see inset to Fig. 2C). (D) The first non-zero Eigen vector from a spectral decomposition of the rate matrix highlighting an exchange in population between folded-like subpopulations (positive amplitude) and disordered states (negative amplitude). (E) The observed rate constants (circles) and their fits (lines) from diffusive simulations on the free energy profiles shown in panel B. (F) The estimated conformational diffusion coefficients of CytR and its variants (lines) compared against the direct experimental measures on select proteins (BBL, λ_6–85_) and that extracted from a WSME model analysis of bACBP folding kinetics (pink circles).

**Fig. 4:**
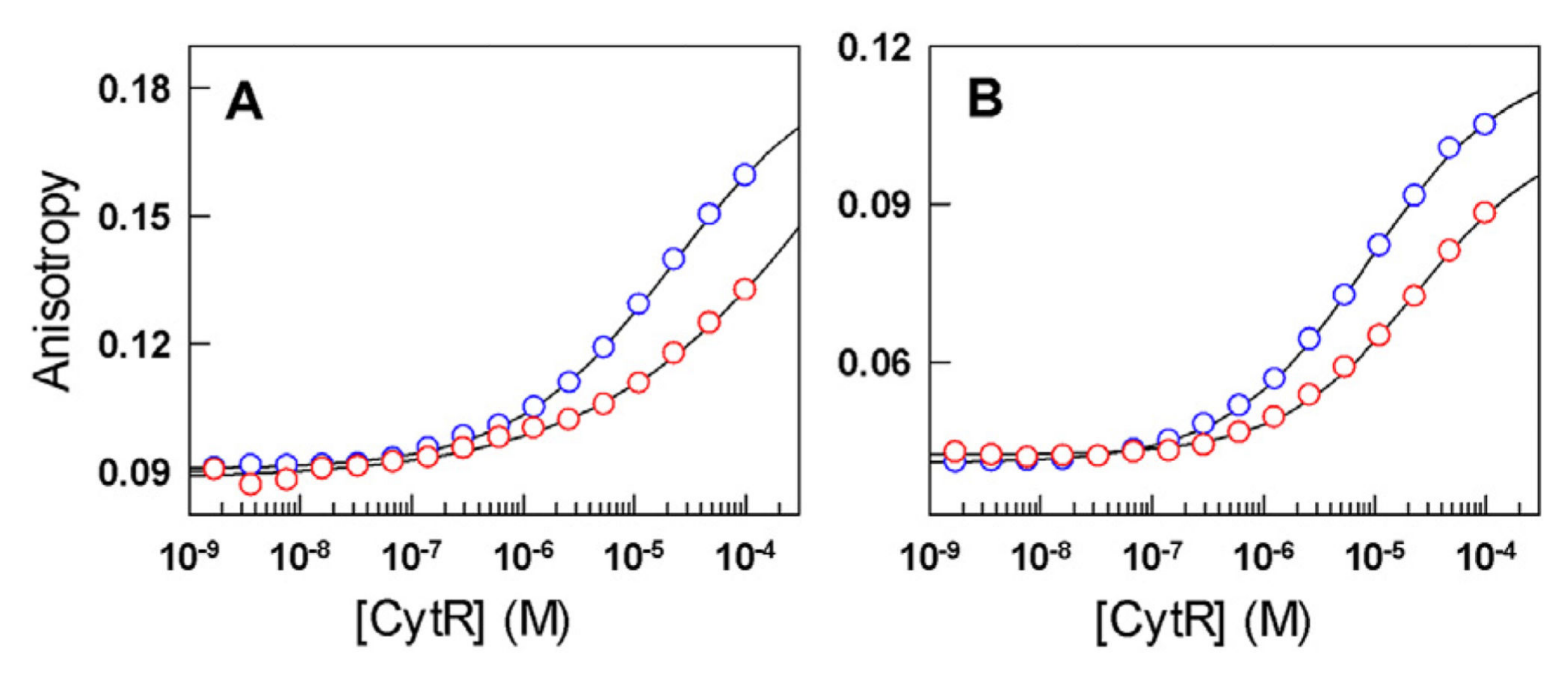
Binding isotherms of CytR (blue) and the P33A mutant (red) to Alexa-532 labeled *udp* promoter at 278 K (A) and 308 K (B). The curves are shown to guide the eye.

## Abbreviations used

IDPs: intrinsically disordered proteins
QY: quantum yield
WSME: Wako–Saitô–Muñoz–Eaton
CD: circular dichroism

## References

[1] Uversky VN, Gillespie JR, Fink AL (2000). Why are “natively unfolded” proteins unstructured under physiologic conditions?. Proteins Struct Funct Genet.

[2] van der Lee R, Buljan M, Lang B, Weatheritt RJ, Daughdrill GW, Dunker AK, Fuxreiter M, Gough J, Gsponer J, Jones DT, Kim PM (2014). Classification of intrinsically disordered regions and proteins. Chem Rev.

[3] Das RK, Ruff KM, Pappu RV (2015). Relating sequence encoded information to form and function of intrinsically disordered proteins. Curr Opin Struct Biol.

[4] Boehr DD, Nussinov R, Wright PE (2009). The role of dynamic conformational ensembles in biomolecular recognition. Nat Chem Biol.

[5] Espinoza-Fonseca LM (2009). Reconciling binding mechanisms of intrinsically disordered proteins. Biochem Biophys Res Commun.

[6] Mollica L, Bessa LM, Hanoulle X, Jensen MR, Blackledge M, Schneider R (2016). Binding mechanisms of intrinsically disordered proteins: theory, simulation, and experiment. Front Mol Biosci.

[7] Bruun SW, Iesmantavicius V, Danielsson J, Poulsen FM (2010). Cooperative formation of native-like tertiary contacts in the ensemble of unfolded states of a four-helix protein. Proc Natl Acad Sci U S A.

[8] Best RB, Hummer G, Eaton WA (2013). Native contacts determine protein folding mechanismsin atomistic simulations. Proc Natl Acad Sci U S A.

[9] Naganathan AN, Orozco M (2011). The native ensemble and folding of a protein molten-globule: functional consequence of downhill folding. J Am Chem Soc.

[10] Iesmantavicius V, Dogan J, Jemth P, Teilum K, Kjaergaard M (2014). Helical propensity in an intrinsically disordered protein accelerates ligand binding. Angew Chem Int Ed.

[11] Muñoz V, Campos LA, Sadqi M (2016). Limited cooperativity in protein folding. Curr Opin Struct Biol.

[12] Banerjee PR, Mitrea DM, Kriwacki RW, Deniz AA (2016). Asymmetric modulation of protein order–disorder transitions by phosphorylation and partner binding. Angew Chem Int Ed Engl.

[13] Banerjee PR, Moosa MM, Deniz AA (2016). Two-dimensional crowding uncovers a hidden conformation of alpha-synuclein. Angew Chem Int Ed Engl.

[14] Nicholson H, Tronrud DE, Becktel WJ, Matthews BW (1992). Analysis of the effectiveness of proline substitutions and glycine replacements in increasing the stability of phage T4 lysozyme. Biopolymers.

[15] Eyles SJ, Gierasch LM (2000). Multiple roles of prolyl residues in structure and folding. J Mol Biol.

[16] Osvath S, Gruebele M (2003). Proline can have opposite effects on fast and slow protein folding phases. Biophys J.

[17] Prajapati RS, Das M, Sreeramulu S, Sirajuddin M, Srinivasan S, Krishnamurthy V, Ranjani R, Ramakrishnan C, Varadarajan R (2007). Thermodynamic effects of proline introduction on protein stability. Proteins.

[18] Elbaum-Garfinkle S, Cobb G, Compton JT, Li XH, Rhoades E (2014). Taumutants bind tubulin heterodimers with enhanced affinity. Proc Natl Acad Sci U S A.

[19] Perez RB, Tischer A, Auton M, Whitten ST (2014). Alanine and proline content modulate global sensitivity to discrete perturbations in disordered proteins. Proteins.

[20] Crabtree MD, Borcherds W, Poosapati A, Shammas SL, Daughdrill GW, Clarke J (2017). Conserved helix-flanking prolines modulate intrinsically disordered protein: target affinity by altering the lifetime of the bound complex. Biochemistry.

[21] Moody CL, Tretyachenko-Ladokhina V, Laue TM, Senear DF, Cocco MJ (2011). Multiple conformations of the cytidine repressor DNA-binding domain coalesce to one upon recognition of a specific DNA surface. Biochemistry.

[22] Munshi S, Gopi S, Subramanian S, Campos LA, Naganathan AN (2018). Protein plasticity driven by disorder and collapse governs the heterogeneous binding of CytR to DNA. Nucleic Acids Res.

[23] Naganathan AN, Orozco M (2013). The conformational landscape of an intrinsically disordered DNA-binding domain of a transcription regulator. J Phys Chem B.

[24] Bryngelson JD, Onuchic JN, Socci ND, Wolynes PG (1995). Funnels, pathways, and the energy landscape of proteinfolding— a synthesis. Proteins.

[25] Naganathan AN, Doshi U, Muñoz V (2007). Protein folding kinetics: Barrier effects in chemical and thermal denaturation experiments. J Am Chem Soc.

[26] Wako H, Saito N (1978). Statistical mechanical theory of protein conformation. 2. Folding pathway for protein. J Phys Soc Jpn.

[27] Muñoz V, Eaton WA (1999). A simple model for calculating the kinetics of protein folding from three-dimensional structures. Proc Natl Acad Sci U S A.

[28] Naganathan AN (2012). Predictions from an ising-like statistical mechanical model on the dynamic and thermodynamic effects of protein surface electrostatics. J Chem Theory Comput.

[29] Felitsky DJ, Record MT (2003). Thermal and urea-induced unfolding of the marginally stable lac repressor DNA-binding domain: a model system for analysis of solute effects on protein processes. Biochemistry.

[30] Lapidus LJ, Steinbach PJ, Eaton WA, Szabo A, Hofrichter J (2002). Effects of chain stiffness on the dynamics of loop formation in polypeptides. Appendix: testing a 1-dimensional diffusion model for peptide dynamics. J Phys Chem B.

[31] DeCamp SJ, Naganathan AN, Waldauer SA, Bakajin O, Lapidus LJ (2009). Direct observation of downhill folding of lambdarepressor in a microfluidic mixer. Biophys J.

[32] Li P, Oliva FY, Naganathan AN, Muñoz V (2009). Dynamics of one-state downhill protein folding. Proc Natl Acad Sci U S A.

[33] Munshi S, Naganathan AN (2015). Imprints of function on the folding landscape: functional role for an intermediate in a conserved eukaryotic binding protein. Phys Chem Chem Phys.

