# Supplementary material for: Entropic Control of an Excited Folded-Like Conformation in a Disordered Protein Ensemble
Source: J Mol Biol. Author manuscript; Available in PMC 2018 Oct 1. (PMC6166778; doi:10.1016/j.jmb.2018.06.008)
Supplement: Supporting Information [file NIHMS78248-supplement-Supporting_Information.pdf]

Supporting Information

Entropic Control of an Excited Folded-Like Conformation in a Disordered Protein Ensemble

Sneha Munshi, Divya Rajendran & Athi N. Naganathan*

Department of Biotechnology, Bhupat & Jyoti Mehta School of Biosciences, Indian Institute of Technology Madras (IITM), Chennai 600036, India.

e-mail: athi@iitm.ac.in

Supporting Methods

Protein Expression, Purification, Equilibrium Unfolding and Binding Studies The purification of CytR DBD and all the equilibrium experiments were performed employing identical protocols to that detailed in a recent work.[1]

Briefly, the equilibrium fluorescence experiments were performed in a Chirascan Plus qCD instrument (Applied Photophysics Ltd., UK) by exciting ∼20-26 μM of CytR in 10 × 10 mm pathlength cuvette at 274 nm and collecting the emission spectra between 280 and 400 nm. The quantum yields were estimated with NATA as a reference (0.13 at 298 K in water). The binding isotherms were generated by exciting Alexa-532 at 530 nm (labeled at the 5'-end of the 14-mer *udp* half-site) and collecting the emission at 580 nm following a 5-minute equilibration for every point in a Chirascan Plus qCD instrument (Applied Photophysics Ltd., UK) equipped with a fluoresence polarization accessory.

Kinetic Measurements The temperature dependent conformational relxation in the disorderd CytR native ensemble was monitored by fluorescence in a Chirascan SF.3 Stopped Flow instrument (Applied Photophysics Ltd., UK; deadtime ~2 milliseconds) coupled to a thermostated water bath. A 200 μM protein solution was denatured in 20 mM sodium phosphate (pH 7.0; native buffer) containing 4 M urea. Refolding was initiated by mixing the protein in urea with the native buffer in a 1:10 ratio (protein:buffer). The relaxation traces were recorded by exciting the sole tyrosine in CytR (Y53) with a 280 nm LED. The total fluorescence was recorded by a PMT detector and the scattered photons were filtered out by placing a 295 nm cut-off filter. Kinetic traces were recorded from 278 to 303 K at an interval of 2.5 K. At each temperature, 6 repeats were recorded while allowing for an equilibration of one minute after every repeat.

Wako-Saitô-Muñoz-Eaton (WSME) Model WSME model invokes an Ising-like treatment to generate protein conformational states (microstates or species) by assuming that each residue can sample two phases, folded (represented by binary variable *1*) and unfolded (binary variable *0*), resulting in an ensemble of 2^N states for a *N*-residue protein.[2,3] The total partition function, partial partition functions, free energy profiles and residue probabilties were calculated as explained in several other works.[2,4-7] The only difference between the simulations employed in the current work and that before[8] is that a sequence-dependent conformational entropy is employed wherein the entropic penalty for fixing P33 in a native conformation is assumed to be zero for CytR. The final paramters that reproduce the unfolding curves are given in Table T1.

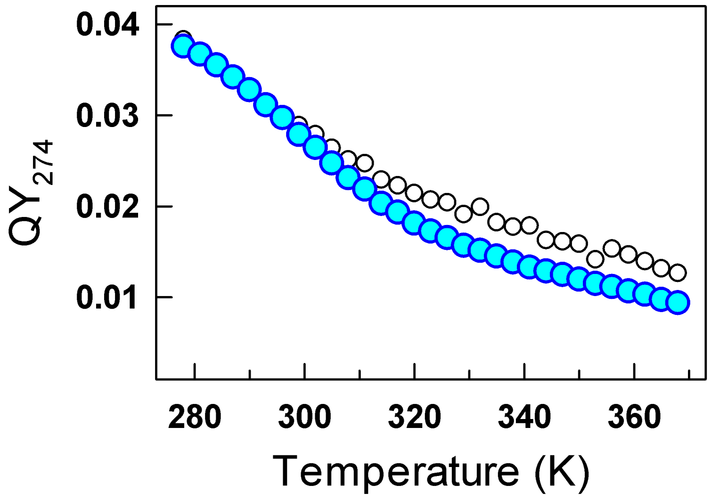

Figure S1 QY of Y53 under native conditions (cyan; 20 mM sodium phosphate buffer, pH 7.0) and in the presence of 6 M urea (black).

```
               10        20        30        40        50        60        70
CytR  MKAKKQETAATMKDVALKAKVSTATVSRALM---NPDKVSQATRNRVEKAAREVGYLPQPMGRNVKRNE--
LacR  MKP------VTLYDVAEYAGVSYQTVSRVVN---QASHVSAKTREKVEAAMAELNYIPNRVAQQLAGKQSL
PurR  --------MATIKDVAKRANVSTTTVSHVIN---KTRFVAEETRNAVWAAIKELHYSPSAVARSLKVN---
FurR  MK---------LDEIARLAGVSRTTASYVINGKAKQYRVSDKTVEKVMAVVREHNYHPNAVAAGLRA----
CcpA  MN-------VTIYDVAREASVSMATVSRVVN---GNPNVKPSTRKKVLETIERLGYRPNAVARGLAS----
DtxR  MKDLVDTTEMYLRTIYELEEEGVTPLRARIA---ERLEQSGPTVSQTVARMERDGLVVVASDRSLQMT---
```

Figure S2 Multiple sequence alignment of CytR homologs. The position 33 (as per CytR numbering scheme) is highlighted in red. Note that only CytR has a proline at position 33 while the others do not.

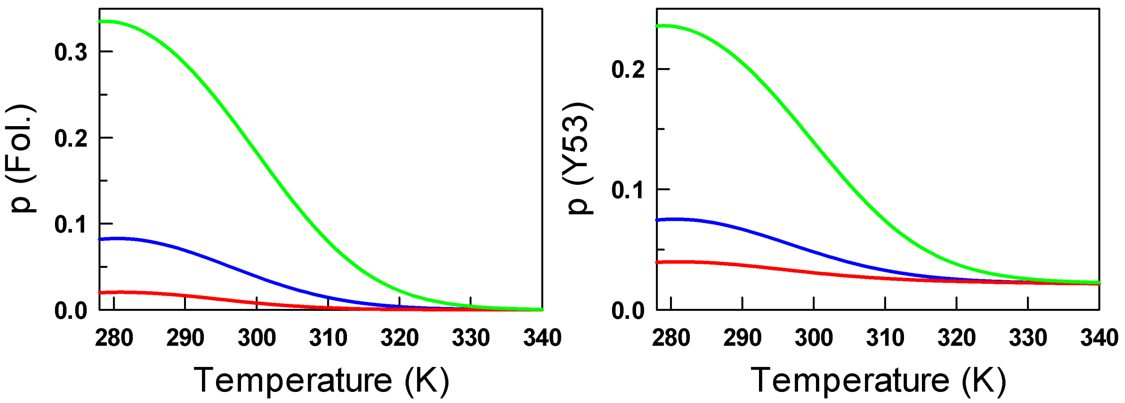

Figure S3 WSME model predictions for CytR (blue), CytR + 0.5 M TMAO (green) and CytR P33A (red). (Left Panel) Population of the folded state. (Right panel) The probability of a folded-like environment for Y53 derived from the partial partition functions.

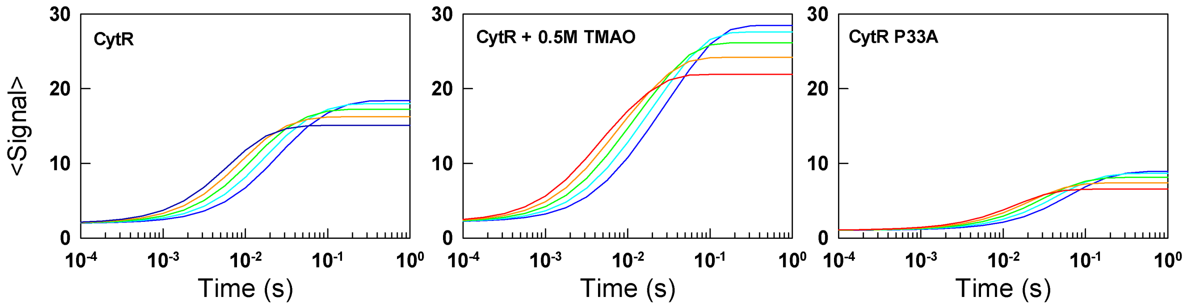

Figure S4 WSME model predictions on the kinetic phases at select temperatures of 283 K (blue), 288 K (cyan), 293 K (green), 298 K (orange) and 303 K (red). Note that in all cases, the amplitude decreases with temperature while the rate increases. The ordering of the overall amplitude is also in agreement with experiments.

Table T1: Parameters* from fits to the experimental far-UV CD data of CytR employing LacR unfolding curve as a reference. The parameters *a/c* and *b/d* are the intercepts and slopes of the folded (*F*) and unfolded (*U*) linear baselines, respectively, in mean residue ellipticity units (MRE; scaled down by a factor of 1000). 298 K is used as the reference temperature for both the linear baselines.

	**ΔS_{conf} ($J\ mol^{-1}\ K^{-1}$)** (all residues except position 33)	**ΔS_{conf} ($J\ mol^{-1}\ K^{-1}$)** (for residue 33)	**ξ ($J\ mol^{-1}$)**	**ΔC_p^{cont} ($J\ mol^{-1}\ K^{-1}$)**	***a***	***b***	***c***	***d***
**CytR**	-34.0	0	-213.0	-2.1	-15.3	0.03	-1.65	-0.04
**CytR + TMAO**	-34.0	0	-216.1	-2.1	-15.3	0.03	-1.65	-0.04
**CytR P33A**	-34.0	-34.0	-213.0	-2.1	-15.3	0.03	-1.65	-0.04

* **ΔS_{conf}** is the entropic penalty for fixing a residue in native conformation. **ξ** is the van der Waals interaction energy per native contact (derived from a 5 Å heavy-atom cut-off radius and including nearest neighbor interactions). ΔC_p^{cont} is the heat-capacity change per native contact.

References

1. Munshi, S., Gopi, S., Subramanian, S., Campos, L.A. & Naganathan, A.N. Protein plasticity driven by disorder and collapse governs the heterogeneous binding of CytR to DNA. *Nuc. Acids Res.*, DOI: 10.1093/nar/gky176 (2018).
2. Wako, H. & Saito, N. Statistical Mechanical Theory of Protein Conformation .2. Folding Pathway for Protein. *J. Phys. Soc. Japan* **44**, 1939-1945 (1978).
3. Muñoz, V. & Eaton, W.A. A simple model for calculating the kinetics of protein folding from three-dimensional structures. *Proc. Natl. Acad. Sci. U.S.A.* **96**, 11311-11316 (1999).
4. Naganathan, A.N. Predictions from an Ising-like Statistical Mechanical Model on the Dynamic and Thermodynamic Effects of Protein Surface Electrostatics. *J. Chem. Theory Comput.* **8**, 4646-4656 (2012).
5. Naganathan, A.N. A Rapid, Ensemble and Free Energy Based Method for Engineering Protein Stabilities. *J. Phys. Chem. B* **117**, 4956-4964 (2013).
6. Naganathan, A.N., Sanchez-Ruiz, J.M., Munshi, S. & Suresh, S. Are Protein Folding Intermediates the Evolutionary Consequence of Functional Constraints? *J. Phys. Chem. B* **119**, 1323-1333 (2015).
7. Rajasekaran, N., Gopi, S., Narayan, A. & Naganathan, A.N. Quantifying Protein Disorder through Measures of Excess Conformational Entropy. *J. Phys. Chem. B* **120**, 4341-4350 (2016).
8. Naganathan, A.N. & Orozco, M. The conformational landscape of an intrinsically disordered DNA-binding domain of a transcription regulator. *J. Phys. Chem. B* **117**, 13842-13850 (2013).
